# Sarcoma of the Dura Mater

**Published:** 1876-11

**Authors:** E. N. Brush


ART. II.—*Sarcoma of the Dura Mater. Report of a Case, with Post Mortem Examination.* By E. N. Brush, M. D.

In March, 1875, I was invited by my friend, Dr. O'Brien, of this city, to see Mr. S., who had sought his advice in reference to a tumor growing upon his head. I found at about the junction of the saggital with the lambdoid suture a tumor about the size of an English walnut. Its growth, I was informed, had been slow and unattended by pain, it was quite movable under the scalp and pretty firm pressure did not cause any unpleasant feeling. I diagnosed a sebaceous tumor of the scalp and advised its removal. Not being able to leave his business at the time, Mr. S. preferred to postpone the operation until he could take a short vacation in the summer.

I saw nothing more of the patient until February 13th, 1876, when I was requested to assist in the removal of the tumor, which I was told, had rapidly increased in size, and was, on that account, somewhat inconvenient. I was considerably surprised at the size and shape which the tumor had now attained. It had extended further back on the head, so that in lying on his back the patient said the tumor was uncomfortably pressed upon. The growth now measured about four and one-half inches in one diameter by from five to six in the other, and projected from the cranium about three inches in the thickest portion. It was but slightly movable and was irregularly nodulated. Its exterior had a shining vascular appearance in some portions which attracted our attention. The immobility we accounted for by the tension produced by the large size of the growth, and to this we also attributed the shining appearance of the scalp. The tumor was not painful on pressure and did not pulsate. It felt soft and elastic. Ether was administered and Dr. O'Brien began the operation by making an incision from before, backwards over the most prominent part of the tumor. We were at once struck by the unusual thickness and great vascularity of the scalp. The scalp seemed dense and its vessels were very much enlarged, an incision at least half an inch deep being necessary before the tumor was reached.

The growth presented itself at the bottom of the cut, having what was, apparently, a containing sac or cyst-wall. I now passed my finger into the wound and began the process of enucleating the tumor. It was easily separated from the scalp, but I was somewhat surprised, on approaching the cranium to find that the membrane covering the tumor was not turned inward under the sides of the growth, but that on the contrary it spread out in all directions upon the skull, making it imposible to raise the tumor from its attachments. Thinking that I might have possibly mistaken a layer of fascia for the investing membrane of tumor, I ruptured this membrane with my finger-nail and passed my finger immediately down upon the bone and thence under the tumor. I immediately recognized the fact that I had passed into the tumor, that it was not sebaceous in character and that beyond the membrane I had just ruptured, it had no retaining sac. Using my finger for a director I enlarged the incision that I could more easily examine the character of the tumor and the extent of its attachments. To do this I passed my finger through the opening which I had made in its investing membrane, down to the base of the tumor. In doing this I came in contact with roughened and eroded bone and in sweeping my finger under the growth to separate it from the skull I was startled by passing into a hole in the cranium. From the fact that the substance of the tumor dipped into this hole it was evident that the growth communicated to some extent with the interior of the cranium. It was now deemed best to discontinue the operation, but before closing the incision, Dr. J. F. Miner was sent for. Being in the vicinity, he arrived shortly and on making a careful examination, expressed his opinion that the tumor arose originally within the cranium, that it had eroded its way through the bone, and its removal was of course impossible. The incision was drawn together by sutures, warm water dressings applied and the patient placed in bed.

On recovering from the ether his mind was clear and active, pulse one hundred, and he complaned but little of pain. A portion of the incision healed by first intention, but the great separation of the external attachment of the tumor which I had made with my finger destroyed its vitality and in a few days it was evident that that its was sloughing out. In a short time I was able to lift out a large portion of the most prominent part of the tumor, and after the removal of a few shreds by suppuration, a red protuberant mass, about the size of a walnut was noticed in the bottom of the cavity. The mass bled easily and pulsated regularly, showing that it arose from, or was connected with, the brain. Beyond keeping the wound clean and the patient free from pain, nothing was necessary. His mind was perfectly clear, he watched his case carefully and noted all the symptoms. The pulse never rose above one hundred and twenty and frequently was as low as eighty. No chill or febrile movement was at any time noticed. His condition continued good until the 8th of March, when he noticed a loss of power and sensation in the right side; this condition increased until it almost amounted to complete hemiplegia; at this time he complained of pain in the head, was disturbed and restless when asleep, but at no time delirious. The hemiplegia, which never became complete, improved to some extent after the fourth or fifth day. Bromide of Potassium in twenty grain doses secured freedom from headache, and, to some extent, quiet sleep. On the second day of the paralysis he found himself unable to evacuate his bladder and the catheter had to be resorted to from that time until his death.

On the night of March 22d, I saw him, in the absence of Dr. O'Brien. He was breathing sterterously at the rate of twelve per minute, his pulse was rapid and feeble. He could only be aroused to respond to questions after considerable effort, but when aroused would recognize those about him and give intelligent replies; he would, however, at once sink again into a semi-comatose condition. Death occured in the forenoon of March 23d.

The following notes of the autopsy are furnished by Dr. W. W. Miner, who was present, and subsequently made a microscopical examination of the tumor.

"AUTOPSY.—The tumor projected like a mound from the upper back part of the head, centering in the median line, half-way in the interparietal suture from frontal to occipital bones. Its base had a diameter of about seven inches; it projected about three inches above the skull; had in its center an open, suppurating cavity about two inches in diameter, with ragged edges, fungating, red, tumid, with base about two inches deep, grayish, sloughing. Weight of tumor estimated at one and one-half pounds. On section of the scalp, the tumor was found to raise up the pericranium from beneath. The scalp was thickened in front but not affected by malignant change, was separable from morbid growth on all sides.

"On separating the tumor from its marginal attachments by dissection and uplifting it from the base, it was encised on a level with the external surface of the skull; the central portion of its base being found continuous with a part inside the cranium, passing through an eroded perforation with very ragged edges of irregularly rounded outline and a diameter of from one and one-half to two inches. The eroding process has affected the border of the perforation for a circle of half an inch breadth about it. A space two and one-half inches in diameter on the external surface of the skull anterior to and something larger than the perforation, shows erosion about one fourth way through the thickness of the bone. Other portions of bone covered by base of tumor are very slightly roughened by inflammatory deposit.

"A circular section of the craninm was removed and is preserved in the Museum of the Medical College. Channels for meningeal vessels running towards the perforation are somewhat abnormally developed on the inner surface of this section. The dura mater was thickening, not adherent; pia injected. The portion of the tumor internal the skull was found situated in the dura mater and extended downwards in the falx cerebri an inch and a half; it would weigh about three ounces. Sections of different portions of the growth showed it to be of a whitish color, of a uniform and somewhat firm consistency, except in the neighborhood of the supporating cavity and free from calcareous or bony deposits. Marks of depression existed upon the lobes of the cerebrum on either side the median line. They were of natural appearance and entirely separate from the growth in the meninges. On incision, however, a cavity of pus extending downwards was found in the central part of the left cerebral lobe which was doubtless due to pressure and was the immediate cause of death.

"The circle of erosion and disintegration is much less on the internal surface of the skull. The erosion, however, being more deeply cut than on the external. It would be difficult to determine from the edge of the perforation, bevelled on both sides, whether the malignant process commenced in the connective tissue on the internal surface of the skull, or external. The erosion has for some time been progressing upon both external and internal surfaces simultaneously. The activity of the malignant process has been mainly external and probably wholly in the soft tissue. There was no adhesion of the tumor to the disintegrated bone beneath it. Osseous erosion was occasioned very markedly on the external surface in the space anterior to the perforation by contact with diseased soft parts.

"Microscopical examinations of the tumor showed its tissue to be of whitish color and to yield abundant clear fluid in which very numerous round cells appeared, of which the tissue itself was mainly composed, intercellular material being scanty and hardly to be recognized. It was accordingly determined to be a sarcomatous tumor of the round-celled variety."

The interesting features of this case are the origin of the growth, its size, and the absence of all brain symptoms until so short a time prior to death.

Similar cases are on record in several of the works upon surgery, and the anatomical characteristics of the growth are mentioned in works upon pathological anatomy. Gross, in his surgery, speaks of these tumors, and mentions two operations for their removal which have been reported, and Hamilton (Principles and Practice of Surgery, page 520), speaking of these growths under the term Fungus of the Dura Mater, says: "The prognosis is unfavorable, but the only remedy is the enlargement of the cranial aperture and a careful dissection of the tumor from the Dura Mater." It hardly seems to me that the attempt to remove a tumor of this character, with a full knowledge of its attachments, would be justifiable. Certainly, no operative measures would have been successful in the case under consideration. On page 581 of Holmes' Principles and Practice of Surgery, will be found illustrations of a case in some respects similar to this one. The tumor, however, arose externally and perforated the skull, and was not malignant in character. The only brain symptoms were occasional attacks of epilepsy.

The difficulty of exactly defining the character of this tumor prior to the operation, and the hope that a report of the case may place others on their guard in diagnosing tumors in this locality, is the object in reporting this case.

